# Clinically Significant Minor Blood Group Antigens amongst North Indian Donor Population

**DOI:** 10.1155/2013/215454

**Published:** 2013-12-09

**Authors:** Divjot Singh Lamba, Ravneet Kaur, Sabita Basu

**Affiliations:** Department of Transfusion Medicine, Block D, Level II, Government Medical College & Hospital, Chandigarh 160030, India

## Abstract

*Background*. Racial differences in blood group antigen distribution are common and may result in striking and interesting findings. These differences in blood group antigen distribution are important due to their influence on the clinical practice of transfusion medicine. *Study Design and Methods*. This is a prospective study, involving 1000 healthy regular repeat voluntary blood donors associated with the department. The clinically significant minor blood group antigens of these donors were studied. *Results*. Out of 1000 healthy regular repeat voluntary blood donors, 93% were D positive and 2.8% were K positive. Amongst the Rh antigens, e was the most common (99%), followed by D (93%), C (85.1%), c (62.3%), and E (21.5%). Within the MNS blood group system, antigen frequency was M (88%), N (57.5%), S (57.8%), and s (87.5%). Within the Duffy blood group system, antigen frequency was Fy^a^ (87.3%) and Fy^b^ (58.3%). *Conclusions*. This data base will help us to prevent alloimmunisation in young females, pregnant women, and patients who are expected to require repeated transfusions in life by providing them with antigen matched blood. Antigen negative blood can also be made available without delay to already alloimmunized multitransfused patients.

## 1. Introduction

A total of 30 blood group systems are recognized by the International Society of Blood Transfusion (ISBT). Nine blood group systems (ABO, Rhesus, Kell, Kidd, Duffy, MNS, P, Lewis, and Lutheran) are considered to be clinically significant as these are known to cause hemolytic transfusion reactions (HTR) and hemolytic disease of fetus and newborn (HDFN) [1–4].

In developing countries like India only ABO and D status of blood donor and recipients are taken into account for compatibility testing. However, the phenotype of clinically significant blood group antigens on the donor red blood cells (RBCs) is required to be known at times when alloimmunization is particularly undesirable, such as in young females, pregnant women, and patients who are expected to require repeated transfusions in life, such as thalassemia or sickle cell disease patients. When selecting blood for transfusion to such patients, it would be useful if we have access to already phenotyped RBCs of donor population so that particular antigen typed blood can be given to such patients to prevent alloimmunization [5]. Furthermore, these are beneficial for already immunized patients if the transfusion is urgent and/or if clinically significant alloantibodies to particular antigen/antigens are present in the patient's serum. In such situations, corresponding antigen negative blood can be given to such recipients without much delay [5].

Racial differences in blood group antigen distributions are common and may result in striking and interesting findings. Very little information is available regarding distribution of various clinically significant minor blood group antigens in northern region of our country. The previous studies are done with limited number donors [6–8]. The present study was done to get an insight of frequency of clinically significant minor blood group antigens amongst regular voluntary blood donors and also to lay foundation of starting a donor database on RBC antigens.

## 2. Material and Methods

This prospective study was conducted in the department of transfusion medicine of a tertiary care hospital after approval by the Institutional Ethics Committee and a written informed consent given by the donors.

Blood samples were collected from 1,000 healthy regular repeat voluntary blood donors (who have donated two or more than two times before) between September 2010 and July 2011. Donors to be studied were arranged group-wise, that is, O positive: 34%, A positive: 22%, B positive: 32%, AB positive: 5%, and negatives: 7% as per the frequency of these blood groups in North Indian population [12, 13]. The clinically significant minor blood antigens of the Rh blood group system—C, c, E, and e; Kell blood group system—K; Duffy blood system—Fy^a^ and Fy^b^; MNS blood group system—M, N, S, and s were studied using DIAMED (Bio-Rad Laboratories, DiaMed Switzerland) gel cards.

## 3. Statistical Analysis

The statistical analysis was carried out using Statistical Package for Social Sciences (SPSS Inc., Chicago, IL, version 15.0 for Windows). Qualitative or categorical variables were described as frequencies and proportions. Proportions for gender were compared using chi-square test. Gene frequencies were calculated using the Hardy Weinberg principle where  *p* + *q* = 1 and *p* = {2 × obs(AA) + obs(Aa)}/2 × {obs(AA) + obs(Aa) + obs(aa)}; thus *q* = 1 − *p*.

## 4. Results

**Figure 1:**
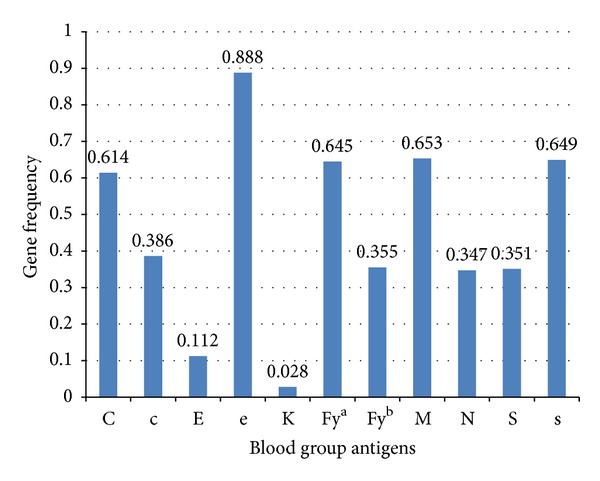
Gene frequency of minor blood group antigens.

The study included 1,000 healthy regular repeat voluntary blood donors of which 947 were males and 53 were females. The males were significantly more than females with *P* < 0.001. The mean age for donors was 35.30 ± 9.86. Of the 1,000 blood donors 930 were D positive and 70 were D negative. The gene frequency for various blood group systems is depicted in Figure 1.

### 4.1. Antigen Frequencies

#### 4.1.1. Rh Antigens

Amongst Rh antigens, e was the most common (99%) followed by D (93%), C (85.1%), c (62.3%), and E (21.5%) (Table 1).

#### 4.1.2. Rh Subgroup Antigens in D Positive and D Negative Donors

98.6% D negative donors had c antigen and 100% D negative donors had e antigen on their red cells. Thus, there is strong association of c antigen and e antigen with D negative donors. C antigen was found to be more associated with presence of D antigen as compared to its absence (90.8% and 10%, resp.) (Table 2).

#### 4.1.3. Minor Blood Group Antigen Frequencies

Amongst minor blood group antigens Kell antigen frequency was 2.8%, Duffy (Fy^a^) 7.3%, Duffy (Fy^b^) 58.3%, M 88%, N 57.5%, S 57.8%, and s 87.5% (Table 3).

### 4.2. Phenotype Frequencies

#### 4.2.1. Duffy Blood Group System

Most common phenotypes in Duffy system were Fy^a^+Fy^b^+ = 45.6% and Fy^a^+Fy^b^− = 41.7% (Table 4).

#### 4.2.2. MNS Blood Group System

Most common phenotypes were M+N− (42.5%), M+N+ (45.5%), S−s+ (42.1%), and S+s+ (45.4%). (Table 5) The frequency of M+N−S+s+, M+N+S+s+, and M+N+S−s+ phenotypes was comparable in our study (Table 6).

## 5. Discussion

Besides ABO and Rh antibodies, antibodies to other clinically significant antigens are also known to cause HTR, HDFN, or shortened survival of transfused red cells [3]. The knowledge of these clinically significant antigens can help in prevention and appropriate management of pregnancies at risk of HDFN and multitransfused patients with alloimmunization. The frequency of such antigens is available for Caucasians and Black races [9–11, 14, 15]. Very limited information is available on antigen and phenotype frequencies in North India. Other studies are either of small sample size or done in particular blood group donors [6–8]. This is the first study where frequency of clinically significant antigens is studied in 1,000 voluntary blood donors. All the antigen and phenotype frequencies reported in our study were compared with that of White and Black population [9–11] and with the other studies from North India [6–8].

In the present study the frequency of D and other Rh antigens was comparable with that of other studies from the region but was markedly different when compared to Whites and Blacks (Table 1).

The antigen frequencies of Duffy and MNS antigens were comparable to antigen frequencies of other studies in this region but were different from that of white and black population (Table 3). The Kell antigen frequency in our study was less (2.8% versus 5.56%) compared to those of another study from this region and is higher than that reported in blacks and in whites (Table 3). The difference in Kell antigen frequency in our study compared to the another study from this region may be due to the fact that donor population in our study includes donors of all blood groups as compared to “O blood group” donors in their study. Thus there is a need to perform more studies with a much larger sample size to know more accurately the antigen frequency of Kell antigen in the population of this region.

The phenotype frequencies of Duffy and MNS blood group systems were compared with those of other studies from the region and with that of white and black population (Tables 4, 5, and 6). These results support the fact that there is variation in the distribution of antigens in the Duffy and MNS blood group system even in North Indian population [7]. Thus there is a need to perform more studies with a much larger sample size to know more accurately the phenotype frequency of Duffy and MNS antigens in the population of this region.

The beta thalassemia carrier rate in India is around 3–7% with higher frequency in northwest India. Approximately 10,000 thalassemia major cases are added each year [16]. The prevalence of alloimmunization in multitransfused patients in India is approximately 3–10% [17, 18]. This study has provided us with donor database of regular repeat voluntary blood donors with known antigenic profile which is referred to, to provide antigen matched blood to young females, pregnant women, and patients who are expected to require repeated transfusions in life. Antigen negative blood is also being provided to already alloimmunized multitransfused patients. This has helped us to prevent alloimmunisation in these groups of patients and prevent already alloimmunised patients from further alloimmunisation.

## 6. Limitations

Kidd antigen evaluation in donors was not done due to cost constraints.

## Figures and Tables

**Table 1:** Comparison of antigen frequency of Rh subgroup antigens.

Antigens	Antigen frequency in total 1000 donors (%)	Antigen frequency in Indians by Thakral et al. [7]	Antigen frequency in Indians by Chaudhary et al. [6]	Antigen frequency in whites [9–11]	Antigen frequency in blacks [9–11]
D	930 (93.0%)	93.4%	ND	85%	92%
C	851 (85.1%)	84.8%	95.2%	68%	27%
c	623 (62.3%)	52.8%	69.2%	80%	96%
E	215 (21.5%)	17.9%	15.4%	29%	22%
e	990 (99.0%)	98.3%	98.1%	98%	98%

**Table 2:** Comparison of antigen frequency of Rh subgroup antigens in D positive and D negative donors.

Antigens	Antigen frequency in D positive donors	Antigen frequency in D negative donors	Antigen frequency in total 1000 donors (%)
C	90.8%	10%	851 (85.1%)
c	59.6%	98.6%	623 (62.3%)
E	22.8%	4.3%	215 (21.5%)
e	98.9%	100%	990 (99.0%)

**Table 3:** Antigen frequency of clinically significant minor blood group antigens.

Blood group antigen	In 1000 cases (%)	Indian study by Thakral et al. [7]	Indian study by Chaudhary et al. [6]	In whites [6]	In blacks [6]
Kell (K)	28 (2.8%)	5.56%	1.92%	0.2%	<0.1%
Fy^a^	873 (87.3%)	86.8%	73.1%	17%	9%
Fy^b^	583 (58.3%)	56.2%	53.8%	34%	22%
M	880 (88%)	75.4%	77.9%	28%	24%
N	575 (57.5%)	61.5%	73.1%	22%	30%
S	578 (57.8%)	56.5%	63.5%	11%	3%
s	875 (87.5%)	87.4%	45.2%	45%	69%

**Table 4:** Phenotype frequencies of Duffy blood group system.

Phenotype	In 1000 cases (%)	Indian study by Thakral et al. [7]	Indian study by Chaudhary et al. [6]	Indian study by Nanu and Thapliyal [8]	In whites [9–11]	In blacks [9–11]
**F** **y** ^**a**^ **+** **F** **y** ^**b**^ **−**	**417 (41.7%)**	**43.9%**	**43%**	**40.8%**	**17%**	**9%**
Fy^a^−Fy^b^+	127 (12.7%)	13.3%	24%	16.2%	34%	22%
**F** **y** ^**a**^ **+** **F** **y** ^**b**^ **+**	**456 (45.6%)**	**42.9%**	**30%**	**42.6%**	**49%**	**1%**
**F** **y** ^**a**^ **−** **F** **y** ^**b**^ **−**	0 (0%)	0%	3%	0.44%	<0.1%	68%

**Table 5:** Phenotype frequencies of MN and Ss in MNS blood group system.

Phenotype	In 1000 cases (%)	Indian study by Thakral et al. [7]	Indian study by Nanu and Thapliyal [8]	In Europeans [14, 15]	In African Americans [14, 15]
**M+N−**	**425 (42.5%) **	**38.5% **	**42.3% **	**28% **	**26% **
M−N+	120 (12.0%)	24.6%	14.6%	22%	30%
**M+N+**	**455 (45.5%)**	**36.9%**	**43.1%**	**50%**	**44%**
S+s−	124 (12.4%)	12.6%	10%	11%	03%
**S−s+**	**421 (42.1%)**	**43.5%**	**62.1%**	**45%**	**69%**
**S+s+**	**454 (45.4%)**	**43.9%**	**26.8%**	**44%**	**28%**
S−s−	1 (0.1%)	0%	1.2%	0%	01%

**Table 6:** Combined phenotype frequencies of MNSs in MNS blood group system.

Phenotype	In 1000 cases (%)	Indian study by Thakral et al. [7]	Indian study by Nanu and Thapliyal [8]	In Europeans [14, 15]	In African Americans [14, 15]
M+N−S+s−	65 (6.5%)	7.9%	5.5%	5.7%	2.1%
**M+N−S+s+**	**208 (20.8%)**	**14.8%**	**13.3%**	**14%**	**7%**
M+N−S−s+	151 (15.1%)	15.8%	22.6%	10.1%	15.5%
M+N+S+s−	50 (5%)	3.5%	4.6%	3.9%	2.2%
**M+N+S+s+**	**201 (20.1%)**	**19.6%**	**10.7%**	**22.4%**	**13%**
**M+N+S−s+**	**204 (20.4%)**	**13.9%**	**27.8%**	**22.6%**	**33.4%**
M−N+S+s−	9 (0.9%)	1.3%	1.2%	0.3%	1.6%
M−N+S+s+	45 (4.5%)	9.5%	3.5%	5.4%	4.5%
M−N+S−s+	66 (6.6%)	13.9%	9.3%	15.6%	19.2%
M−N+S−s−	0 (0%)	0%	0.3%	0%	0.7%
